# Development of a Soil Organic Matter Content Prediction Model Based on Supervised Learning Using Vis-NIR/SWIR Spectroscopy

**DOI:** 10.3390/s22145129

**Published:** 2022-07-08

**Authors:** Min-Jee Kim, Hye-In Lee, Jae-Hyun Choi, Kyoung Jae Lim, Changyeun Mo

**Affiliations:** 1 Agriculture and Life Sciences Research Institute, Kangwon National University, Chuncheon 24314, Korea; kim91618@kangwon.ac.kr; 2 Interdisciplinary Program in Smart Agriculture, Kangwon National University, Chuncheon 24314, Korea; leein31@kangwon.ac.kr; 3 Department of Biosystems Engineering, College of Agriculture and Life Sciences, Kangwon National University, Chuncheon 24314, Korea; jhyun9609@naver.com; 4 Department of Regional lnfrastructure Engineering, College of Agriculture and Life Sciences, Kangwon National University, Chuncheon 24314, Korea; kjlim@kangwon.ac.kr

**Keywords:** topsoil, reflectance spectroscopy, soil organic matter, partial least square regression, support vector machine regression

## Abstract

In the current scenario of anthropogenic climate change, carbon credit security is becoming increasingly important worldwide. Topsoil is the terrestrial ecosystem component with the largest carbon sequestration capacity. Since soil organic matter (SOM), which is mostly composed of organic carbon, and can be affected by rainfall, cultivation, and pollutant inflow, predicting SOM content through regular monitoring is necessary to secure a stable carbon sink. In addition, topsoil in the Republic of Korea is vulnerable to erosion due to climate, topography, and natural and anthropogenic causes, which is also a serious issue worldwide. To mitigate topsoil erosion, establish an efficient topsoil management system, and maximize topsoil utilization, it is necessary to construct a database or gather data for the construction of a database of topsoil environmental factors and topsoil composition. Spectroscopic techniques have been used in recent studies to rapidly measure topsoil composition. In this study, we investigated the spectral characteristics of the topsoil from four major rivers in the Republic of Korea and developed a machine learning-based SOM content prediction model using spectroscopic techniques. A total of 138 topsoil samples were collected from the waterfront area and drinking water protection zone of each river. The reflection spectrum was measured under the condition of an exposure time of 136 ms using a spectroradiometer (Fieldspec4, ASD Inc., Alpharetta, GA, USA). The reflection spectrum was measured three times in wavelengths ranging from 350 to 2500 nm. To predict the SOM content, partial least squares regression and support vector regression were used. The performance of each model was evaluated through the coefficient of determination (R^2^) and root mean square error. The result of the SOM content prediction model for the total topsoil was R^2^ = 0.706. Our findings identified the important wavelength of SOM in topsoil using spectroscopic technology and confirmed the predictability of the SOM content. These results could be used for the construction of a national topsoil database.

## 1. Introduction

Topsoil is rich in organic matter and microorganisms which perform important ecosystem functions, such as pollutant purification, carbon storage, and material recirculation. The Republic of Korea is very vulnerable to erosion of topsoil due to climate, topography, and natural and anthropogenic causes; it is internationally known that topsoil erosion is a serious issue here [1]. Studies on topsoil in the Republic of Korea mainly focus on erosion, examining the amount of topsoil, loss of topsoil via erosion, and improvement of erosion models [2,3,4]. However, to solve the problem of topsoil erosion, establish an efficient management system for topsoil, and maximize the value of topsoil utilization, it is necessary to construct a database or gather data for the construction of a database of topsoil environmental factors. Specifically, topsoil environmental factors that could be changed by rainfall, cultivation, and an inflow of pollutants should be measured and estimated through regular monitoring and modeling [5,6]. Environmental factors of topsoil include carbon, nitrogen, vegetative nutrients, soil texture, trace elements, pH, and soil moisture. Among them, soil organic matter (SOM) plays an important role when evaluating soil erosion, conservation, and quality because it considers factors such as microbial activity, biodiversity, topsoil erosion, and the adsorption of pollutants as well as physical factors such as air and water mobility, and water retention capacity [7,8,9]. The major component in SOM is carbon, which is closely related to the bulk density and cation exchange capacity of the topsoil. The incorporation of organic carbon into topsoil is one of the mechanisms whereby carbon dioxide from the atmosphere is sequestered and is gaining recognition for its role in climate change [6,10,11]. Therefore, investigating SOM content can provide essential environmental data for establishing a topsoil management system and mitigating the effects of global warming.

Existing physicochemical analyses of topsoil components are time consuming, require physical labor, and can be costly depending on the level of precision required, number of samples, and analysis items. To solve these problems, various methods, including electrical resistivity, ion electrode, digital image processing, and spectroscopic technologies, have been used for the analysis of topsoil components [12,13]. Spectroscopic technology can rapidly and non-destructively predict the composition of food, agriculture, and the environment. Visible and near-infrared (Vis-NIR) reflectance spectroscopy can measure the intensity of reflected energy for each wavelength after irradiating sample surfaces [6]. Furthermore, it can detect specific spectra from various substances in the corresponding wavelength band (350–2500 nm) using intrinsic components and can be applied for the qualitative and quantitative analysis of soil components in combination with algorithms such as partial least square regression (PLSR), partial least square discriminant analysis (PLS-DA), support vector regression (SVR), and artificial neural networks (ANN) [9,14,15,16]. However, when using Vis-NIR reflectance spectroscopy in topsoil, a generally complex environmental component, it is difficult to distinguish the absorption patterns of specific components within it because of overlapping absorption and low concentrations of topsoil components [17]. For this reason, analyzing soil spectral data and selecting an appropriate pre-processing technique and algorithm is a key step in improving the performance of soil composition prediction models [16]. Pre-processing techniques are used to remove and minimize noise in soil spectral data and intensify the signal, thereby improving the calibration model. These techniques are mathematical procedures that can transform reflectance measurements, eliminate variability in light scattering effects, and improve spectral features [18]. In many previous studies, predictive models of SOM, carbon, and nitrogen were developed using the PLSR model, which extracted complex absorption patterns and demonstrated high levels of efficiency when investigating a correlation between patterns and soil [17,19,20,21,22]. Owing to the relationship between spectral data and soil characteristics rarely being linear, studies applying neural networks and machine learning-based ANN, SVR, and random forest have been conducted [5,23,24]. Although Vis-NIR spectroscopy is considered a well-established method for estimating soil properties, no accepted universal model currently exists that is widely applied [18]. To develop a standard for predicting soil carbon using a spectroscopic technique, more research should be conducted in terms of selecting a suitable range of the spectral sensor, pre-processing, and calibration technique.

Therefore, the purpose of this study is to develop a model using Vis-NIR spectroscopy and machine learning to construct a SOM database of the environmental factors of the topsoil, and to rapidly measure SOM components. More specifically, we collected topsoil samples from the basins of four major rivers in the Republic of Korea and investigated the characteristics of their SOM spectrum. Furthermore, SOM prediction PLSR and SVR models, to which various spectral pre-processing methods was applied for three wavelength ranges (400–1100 nm, 1100–2500 nm, and 350–2500 nm), were developed and compared to determine the optimal SOM prediction model.

## 2. Materials and Methods

**Figure 1:**
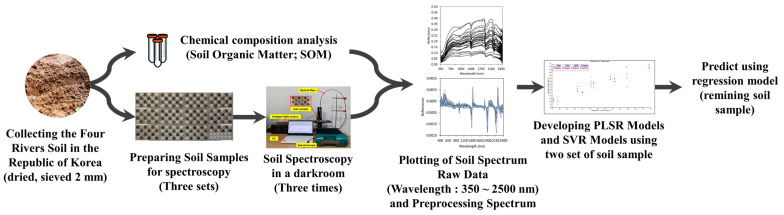
Experimental flowchart for developing SOM prediction models.

This experiment was performed in the order shown in Figure 1. Topsoil samples were collected from the basins of four major rivers in the Republic of Korea, brought to the laboratory, and pretreated by air drying and sorting. Chemical composition analysis was performed on the pretreated topsoil samples which were then placed on 6 × 4 well multi-dishes for spectral measurement. After the spectral analysis was performed in a dark room, the correlation between the chemical composition analysis and the spectrum of topsoil samples was analyzed. Subsequently, a machine learning model for predicting the SOM content was developed and the performance of the models was evaluated using verification steps. The specific experimental methods are described in Figure 1 below.

### 2.1. Study Region and Soil Samples

**Figure 2:**
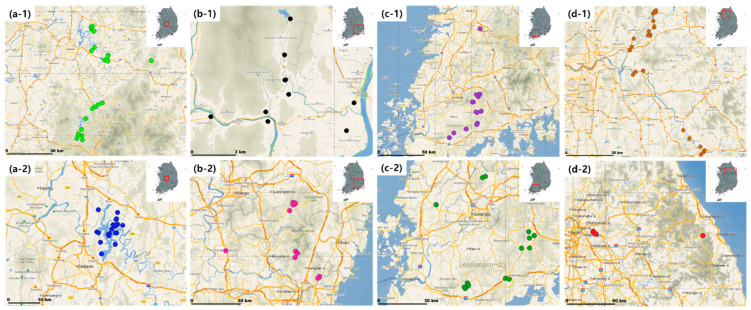
Study area with topsoil samples’ locations (the alphabet means the four major rivers in the Republic of Korea: (**a**) Geum River, (**b**) Nakdong River, (**c**) Yeongsan/Seomjin River, and (**d**) Han River. The waterfront area and drinking water protection zone are represented by (**1**) and (**2**) respectively).

The topsoil samples were collected from 138 points in the waterfront area (WA) and drinking water protection zone (DWPZ) of four major rivers (Geum River; GR, Nakdong River; NR, Yeongsan/Seomjin River; YSR, Han River; HR) in the Republic of Korea in 2019. The locations where topsoil samples were collected are shown in Figure 2. Topsoil samples (50 g each) were collected using a hand auger, and samples from five points in each area were mixed and homogenized as representative samples. The collected topsoil samples were brought to the laboratory, air-dried, and filtered through a 2 mm sieve to remove coarse roots and rock fragments. The SOM of each topsoil sample was determined in the laboratory using the Walkley-Black method. The SOM was measured at a wavelength of 610 nm with an ultraviolet/visible spectrophotometer (UV-2401PC, Shimadzu, Kyoto, Japan) by adding distilled water after oxidizing the soil by adding potassium dichromate and sulfuric acid [25].

### 2.2. Spectral Measurements

**Figure 3:**
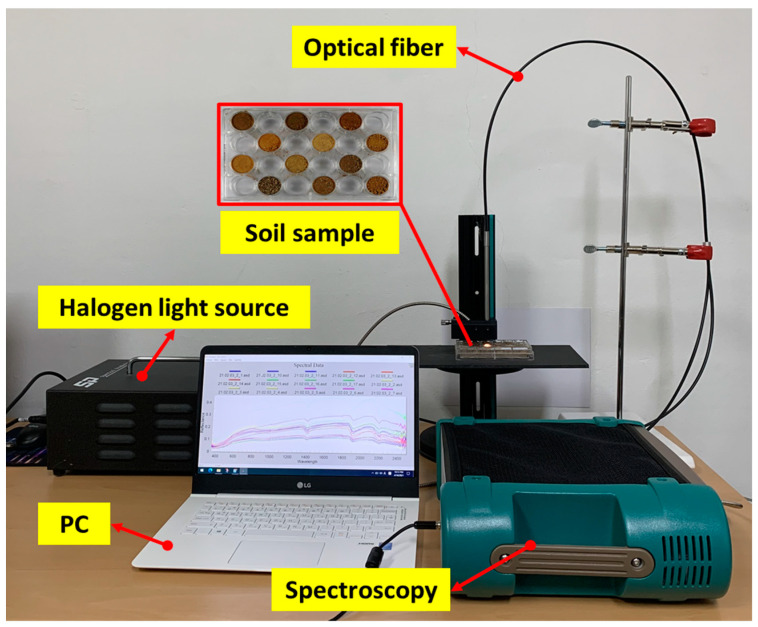
Experimental equipment setup for spectral spectrum acquisition of topsoil samples.

The topsoil sample was placed in a 6 × 4 well multi-dish with a radius of 15.85 mm at a thickness of 15.5 mm to measure the reflection spectrum of the topsoil. After removing gaps between particles by applying a constant pressure, the topsoil sample was flattened to obtain a smooth surface, and a total of three sets were composed. The experimental configuration for the spectroscopic experiment is shown in Figure 3. The experiment carried out in a dark room to minimize noise and error of the spectrum during spectral measuring. Furthermore, the distance between the spectroscopic probe and the topsoil sample surface was fixed at 4 mm and a 45° zenith angle. A 100 W tungsten halogen lamp (ASBN-W, Korea spectral products, Seoul, Republic of Korea) was used as a light source and optical fiber was connected to uniformly irradiate the sample surface with incident energy. The reflectance spectra of topsoil samples were obtained using a spectroradiometer (Fieldspec4, ASD Inc., Alpharetta, GA, USA) at a wavelength of 350 to 2500 nm with a spectral resolution of 3 nm at 700 nm and 10 nm at 1400 and 2100 nm. The spectroradiometer has a bandwidth of 1.4 nm at 350 to 1000 nm and 1.1 nm at 1001 to 2500 nm. Spectra were acquired under the condition of an exposure time of 136 ms and recorded with a sampling resolution of 1 nm to obtain reflectance of 2151 wavelength. The spectra reflectance of each topsoil sample was measured after scanning each sample ten times. For each topsoil sample, measurements were repeated three times, and 414 spectral data were obtained from one set of topsoil samples. The reflectance of the topsoil samples was adjusted by using dark and white references to correct the device noise. The reflectance of the dark reference was obtained without a light source and the white reference used diffused reflectance standards (Labsphere, North Sutton, NH, USA).

### 2.3. Spectral Pre-Processing

Spectral pre-processing techniques can correct shape distortion, light scattering, and noise of a spectrum that may be generated in an external environment [26,27]. In this study, various pre-processing techniques were applied and evaluated to improve the performance of the topsoil SOM prediction model.

Pre-processing was conducted using the average spectrum of each topsoil sample to determine the optimal conditions for the prediction of SOM. The spectra pre-processing techniques were applied, including smoothing with moving average (5 nm), maximum normalization, Savitzky–Golay first-order derivatives, Savitzky–Golay second-order derivatives, multiplicative scatter correction (MSC), and the standard normal variate (SNV). The performances of the SOM prediction models for each pre-processing method were compared and evaluated. Pre-processing using the derivatives method was performed at 10, 15, and 20 nm intervals. The Unscrambler X (v.10.4, CAMO Software, Oslo, Norway) was used for spectral pre-processing.

### 2.4. Multivariate Data Methods

PLSR and SVR, both multivariate techniques, were used to analyze topsoil characteristics using spectral data. The PLSR model is a method used in various applications such as spectral data analysis. PLSR aims to find linear combinations that account for variations of predictor (x, spectrum) and response variables (y, soil properties) that describe the common structure [28]. To maximize the covariance between x and y, the PLSR algorithm incorporates successive regression and compression steps to obtain a set of orthogonal factors called latent variables (LVs). The validation method of leave-one-out cross-validation (LOOCV) was used to evaluate the PLSR model for the calibration set [17,23]. Optimal factors for PLSR models were obtained by minimizing root mean square error of cross-validation (RMSECV), using the LOOCV technique [29,30].

Regression coefficients (b-coefficients) were applied to determine the effective wavelength band from the PLSR calibration. When the b-coefficient value exceeded the thresholds, which were set to the standard deviation of their values, the corresponding wavelength was considered significant [17,23,31]. 

Support vector machines (SVM) are machine learning methods based on statistical learning. SVR, an application of SVM for regression models, can map input variables into high-dimensional feature spaces by applying kernel functions [31]. The SVR model has the advantage of developing model predictions with small samples. In this study, the SOM prediction SVR model was developed by applying a radial basis function kernel.

Table 1 shows the number of data sets used for model development and prediction. Three independent sample set groups were constructed from 138 topsoil samples to develop and predict the PLSR and SVR models. The data set included two subsets: a calibration data set used for developing the SOM prediction model (two sample set groups), and an independent test subset applied to assess the prediction model (remaining test set). Models were developed for the total topsoil sample (138 samples) and each of the four major rivers in the three-level wavelength range of 400–1100 nm (Vis-NIR), 1100–2500 nm (shortwave near-infrared; SWIR), and 350–2500 nm. The three-level wavelength range was set based on the experimental designs of previous studies [18,26,27,32]. In general, the measurement spectrum band is a major factor determining the application field of the spectroscopic technique. Vis-NIR wavelength band and SWIR wavelength band are mainly used in atmospheric environment and surface soil applications [33]. Soils in each of the four major rivers were classified into total, WA, and DWPZ to develop a model. The WA and DWPZ of the four major rivers in Republic of Korea are sensitive to water quality and surrounding land use, and can represent differences in soil composition; thus, a model was developed by classifying area [34]. The PLSR and SVR models were developed using The Unscrambler X (v.10.4, CAMO Software, Oslo, Norway).

### 2.5. Model Validation

The actual SOM values of topsoil were compared with those predicted from the calibration (cross-validation) or independent validation data sets using the PLSR and SVR models. The performance of each developed PLS and SVR model was evaluated using the coefficient of determination (R^2^), root mean square error of calibration (RMSE_C_), root mean square error of validation (RMSE_V_), and optimal factor (F). The R^2^ and RMSE were calculated according to Equations (1) and (2), respectively [35]. The biases of the predictions in the calibration and validation sets were also calculated using Equation (3). Validation of the developed model was performed on unknown topsoil samples by selecting the model with the highest SOM predictability under each condition from the evaluated models. The predictive model’s performance was compared and analyzed through R^2^ and RMSE.

(1) $$R^{2}=1-\frac{\sum_{i=1}^{n}{\left(y_{i}-{\hat{y}}_{i}\right)}^{2}}{\sum_{i=1}^{n}{\left(y_{i}-{\bar{y}}_{i}\right)}^{2}}$$

(2) $$RMSE=\sqrt{\frac{\sum_{i=1}^{n}{\left(y_{i}-{\hat{y}}_{i}\right)}^{2}}{N}}$$

(3) $$\mathrm{bias}=\overset{n}{\underset{i=1}{\sum }}\frac{\left({\hat{y}}_{i}-y_{i}\right)}{N}$$

where $y_{i}$ and ${\hat{y}}_{i}$ are the reference and predicted values of target variables in the $i^{th}$ sample, respectively; The ${\bar{y}}_{i}$ is the mean of reference values, while N is the number of samples.

## 3. Results and Discussion

### 3.1. SOM Content of Topsoil Samples

SOM is an index that can evaluate the soil’s organic carbon holding capacity. Table 2 shows the minimum (Min.), maximum (Max.), average (Ave.), and standard deviation (Std.) of SOM content in each of the four major rivers. The SOM content of all 138 topsoil samples ranged from 8.00 to 77.03 g kg^−1^, with an average value of 32.44 g kg^−1^. As a result of measuring the SOM content for each of the four major rivers, the average value was found for each: NR (52.61 g kg^−1^), YSR (37.83 g kg^−1^), GR (27.39 g kg^−1^), and HR (17.44 g kg^−1^). In the case of WA, the average SOM value was the highest in YSR (39.06 g kg^−1^) compared to other rivers. The highest average SOM content in the DWPZ was in the NR (61.80 g kg^−1^). The standard deviation was lower in the HR than in the other rivers for the total, WA, and DWPZ. Statistical processing (where the SOM component of each of the four major rivers was divided into WA and DWPZ) revealed a significant difference in the average SOM content of NR. For the remaining rivers, the SOM content of WA had a wider range than that of DWPZ. The range of SOM content in WA of YSR was the widest (10.82 to 77.03 g kg^−1^) and the standard deviation was the highest (19.56 g kg^−1^).

### 3.2. Spectral Soil Properties

**Figure 4:**
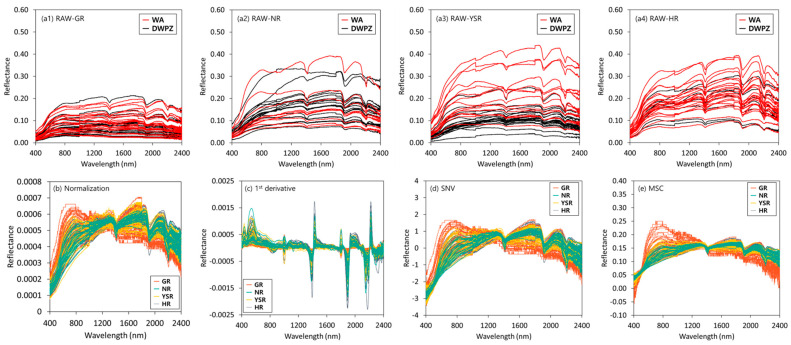
(**a**) Raw reflection spectrum of topsoil samples in (**a1**) Geum River, (**a2**) Nakdong River, (**a3**) Yeongsan/Seomjin River, and (**a4**) Han River and reflection spectrum with applied main pre-processing ((**b**) maximum normalization, (**c**) 1st derivative, (**d**) SNV, and (**e**) MSC).

The raw reflection spectrum of the topsoil samples and the reflection spectrum applying the major pre-processing technique are illustrated in Figure 4. In the case of the spectrum without pre-processing, a difference in reflectivity was observed depending on the collection location of the topsoil sample. The reflectance in GR was lower than that of other rivers. However, overall similar spectral characteristics were shown in the four major rivers. In the wavelength range between 400 and 750 nm, a sharp reflectance gradient was observed, and relatively strong absorption appeared around 1400, 1910, and 2200 nm. Generally, SOM appears distinctly in the NIR region because of the presence of chemical bonds such as C-H (aliphatic), C-H (aromatic), C-O (carboxyl), O-H (hydroxyl), and N-H (amine and amide) [36,37]. A previous study reported that 1400 and 1900 nm are water-related wavelength regions, the absorption region at 1400 nm is the first overtone of O-H stretching (moisture adsorbed to the topsoil surface), and the ration absorption region at approximately 1900 nm is the combination of O-H stretching and H-O-H bending of trapped water molecules in the crystal lattice [23]. However, it was recently reported that in the case of an air-dried soil sample, a peak at around 1400 nm might occur because of the first overtone of C-H functional groups related to organic matter [17]. A spectral absorption peak of approximately 2200 nm depended on phenolic O-H, amide N-H, amine N-H, and aliphatic C-H SOM groups [20,23,38]. 

As a result of pre-processing the spectrum, similar spectral appearances were observed when the (b) maximum normalization, (d) SNV, and (e) MSC techniques that are used for light scattering correction were applied. In the wavelength range between 400–750 nm, pre-processing showed a sharper reflectance gradient than the spectrum when no pre-processing was applied. Compared to the other rivers, GR shows a sharper reflectance gradient and a strong spectral absorption peak at approximately 750 nm. When the (c) Savitzky–Golay first-order derivatives were applied, strong spectral absorption peaks appeared approximately at 435, 552, 1000, 1393, 1800, 2150 to 2170, and 2325 nm; however, these did not appear when no pre-processing was applied.

### 3.3. PLSR and SVR Model Development in Calibration-Validation Approaches

A total of 468 SOM prediction models, including PLSR models (234 types) and SVR models (234 types), were developed for predicting SOM content in topsoil samples. These were developed by applying 6 spectral pre-processing methods in 3 wavelength ranges. The optimal SOM prediction model was determined by comparing the prediction performance (R_v_^2^ and RMSE_v_ values) of each PLSR and SVR model (Table 3). For each optimal model, the wavelength band and pre-processing technique were determined. Among the prediction models developed for the total topsoil, R_v_^2^ and RMSE_v_ values of SVR were of higher performance compared to the PLSR (R_v_^2^ = 0.630, RMSE_v_ = 11.66 g kg^−1^), at 0.678 and 11.12 g kg^−1^, respectively, when Savitzky–Golay first-order derivatives pre-processing was applied in 350–2500 nm. The optimal number of factors in the PLSR models was between 4 and 11, and the largest optimal number of factors (11) was in the DWPZ of YSR. When comparing models for each river, total topsoil of GR showed lower R_c_^2^ (R_c_^2^ of PLSR = 0.668 and R_c_^2^ of SVR = 0.627) and higher RMSE_c_ values (RMSE_c_ of PLSR = 7.47 g kg^−1^ and RMSE_c_ of SVR = 8.54 g kg^−1^). According to the results of previous studies [6,35], the wavelength band of 400–700 nm is important in predicting the carbon and nitrogen components of the soil. When SNV and maximum normalization pre-processing were applied, the soil peak of GR showed a tendency to increase sharply in the 400–700 nm range. This indicates that this spectral characteristic (overlap of chemical bonding information) acts as a variable in the SOM prediction model at GR.

When developing the model with a wavelength range of 350 to 2500 nm, it was found that the SOM prediction performance was higher than when the model was developed by dividing the wavelength range into 400–1100 nm (Vis-NIR), and 1100–2500 nm (SWIR). Results showed that the various pre-processing techniques had considerable effects on the performance of SOM content prediction models. The performance of the model was improved with the pre-processing of maximum normalization, Savitzky–Golay first-order derivatives, SNV, and MSC. A higher R_v_^2^ value was obtained from the model which classified data as being from WA and DWPZ areas of the four major rivers, than from the model which did not distinguish between data from WA and DWPZ.

### 3.4. Selection of Effective Wavelengths in the PLSR Model 

**Figure 5:**
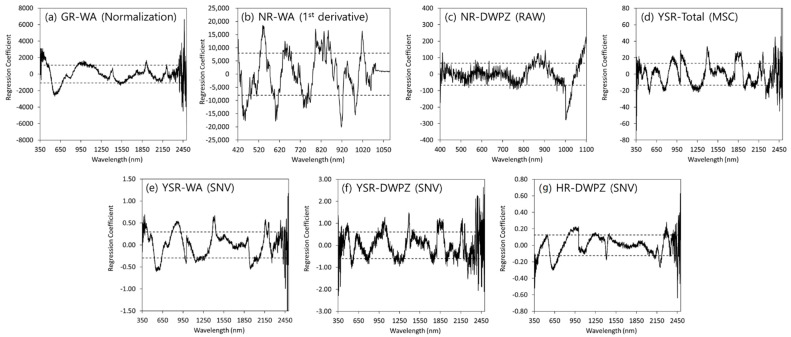
Plots of the regression coefficient with the PLSR models for SOM predictions in (**a**) WA of GR, (**b**) WA of NR, (**c**) DWPZ of NR, (**d**) total of YSR, (**e**) WA of YSR, (**f**) DWPZ of YSR, and (**g**) DWPZ of HR.

The regression coefficient distribution that could determine the effective wavelength in the PLSR model when the optimal model for predicting SOM in the four major rivers was PLSR is shown in Figure 5. The extent of the regression coefficient (positive or negative) represents the importance of the wavelength in explaining the variation in soil properties [23]. The positive or negative regression coefficients were determined to be effective wavelengths that explained the variation of SOM when it exceeded the threshold (dash line) set as the standard deviation. The peak values were different for each selected PLSR model, and in the case of the WA of GR (Figure 5a), a negative peak was observed at 573 nm. The optimal models in the WA and DWPZ of NR were selected in the Vis-NIR region (400–1100 nm). The PLSR model in the WA of NR had significant positive peaks for SOM at approximately 545, 793–854, and 1017 nm and negative peaks at around 455, 607, 917, and 984 nm (Figure 5b). The PLSR model in the DWPZ of NR had a sharp negative peak at approximately 1003 nm (Figure 5c). In the case of the PLSR in the YSR (Figure 5d–f), the regression coefficient at 545, 892, 1002, and 1881 nm were significant in the PLSR model. The significant regression coefficients were shown at approximately 594, 872, 1413, 1943, and 2165 nm using the PLSR model in WA of YSR. The PLSR model in the DWPZ of YSR had significant positive peaks at approximately 483, 1035, 1393, 1844, and 2173 nm and negative peaks at around 552 nm. In the case of the PLSR model in the DWPZ of HR (Figure 5g), the positive regression coefficient peaks at 989 and 2293 nm and negative regression coefficient peaks at 636 and 2233 nm were shown. A previous study reported that, overtones and combination bands of organic molecular compounds occurred through stretching and bending of NH, CH, and CO groups in the Vis-NIR region [17]. In this study, a regression coefficient appeared under the 1100 nm mark. In addition, the results of this study were consistent with those of previous studies which reported that 1700 nm, 2050 nm, and 2426 nm were the main wavelengths for SOM identification [23,38].

### 3.5. Evaluation of Optimal Models’ Performance for Predictive SOM Contents

**Figure 6:**
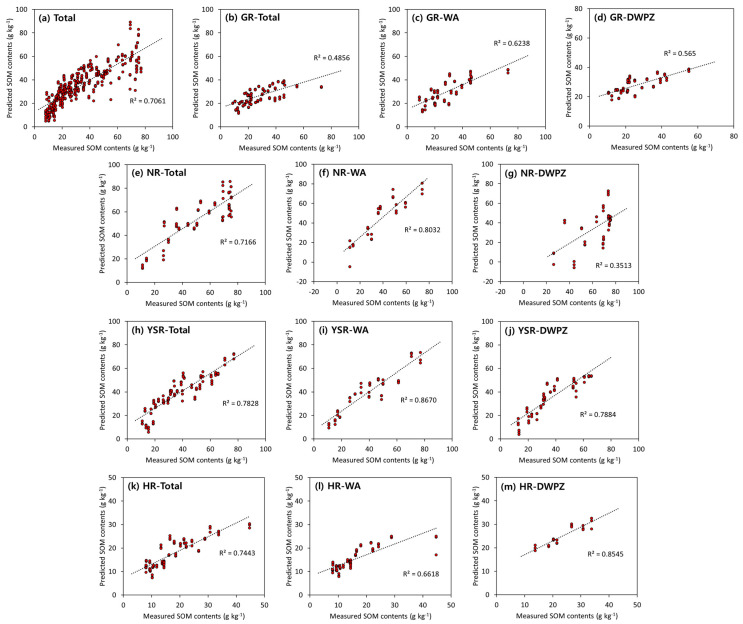
Scatter plot of predicted vs. measured SOM in the prediction dataset for the optimal model in each of the four major rivers (**a**–**m**) built based on calibration set.

The developed optimal SOM prediction model for each of the four major rivers was verified using an unknown topsoil sample as a prediction dataset (Figure 6). For the optimal model of total topsoil (SVR with 1st derivative pre-processing), the R^2^ value was higher than R_v_^2^ (R^2^ = 0.706). Furthermore, as the SOM content increased, the distribution of SOM prediction tended to expand (Figure 6a). The R^2^ value of the SOM prediction model in GR ranged between 0.486 to 0.624 (Figure 6b–d). Among them, the model in DWPZ gave an R^2^ value after validation of 0.565, which was lower than R_v_^2^ (0.735). Overall, the SOM prediction model in GR appears to have a low performance because of weak reflectance due to the overlapping of various components in the topsoil. The prediction R^2^ of the model in NR reached a range of 0.351 to 0.803 (Figure 6e–g). The performance of the model in DWPZ was rather low, at 0.351. The average SOM content of the DWPZ of NR was 61.80 g kg^−1^, and the higher the SOM content in the model verification of the total topsoil (Figure 6a), the wider the predictive distribution and the lower the performance. Therefore, it was judged that the R^2^ value was low owing to the high SOM content in DWPZ of NR. The R^2^ of the SOM prediction model in YSR and HR was found to be similar or higher value than that of the R_v_^2^ value (Figure 6h–m).

## 4. Conclusions

This study focused on different approaches of integrating Vis-NIR and SWIR spectral information for SOM quantification. We identified the characteristics of topsoil, and an optimal wavelength band was determined to develop a model for predicting SOM content in four major rivers in the Republic of Korea. In addition, we developed PLSR and SVR models by applying a pre-processing technique that minimized the discriminant error caused by spectral overlap and improved the model’s performance. The optimal model was determined by applying six spectral pre-processing methods in three types of wavelength ranges to find the optimal condition for high-performance of SOM content prediction.

The SOM content (with overall limited SOM contents ranging from 8.00 to 77.03 g kg^−1^) prediction model using spectral reflectance showed a high prediction performance in the wavelength range between 350 to 2500 nm. It was verified that the combination of the two spectral ranges of the spectrum (Vis-NIR and SWIR) enhanced the performance of the models, resulting in lower errors in the predictions of the SOM components. This is due to the sum of the different information contained in each spectral range related to the SOM component of each topsoil. Moreover, we found that the performance of the SOM prediction model could be improved by applying the maximum normalization, Savitzky–Golay first-order derivatives, SNV, and MSC pre-processing techniques.

In the Republic of Korea, topsoil is vulnerable to erosion due to climatic and topographical factors. Continuous and rapid monitoring is required to prevent this erosion, however, no means to realize this currently exists. When the SOM prediction model was developed without classifying the soil into four categories (the four major rivers), the SOM prediction performance was found to be more 0.7. This finding suggests that useful predictions about the chemical properties of the topsoil can be obtained using spectroscopic techniques. However, with only 138 independent samples, the current model is not yet very robust. Furthermore, when the SOM model was applied to each river, a significant difference in model performance was observed. As the SOM content in the model increased, the performance of the model decreased. In future studies, a robust models should be developed by constructing additional data and applying the optimal multivariate correction technique.

In this study, we identified the important wavelength of SOM (main spectral peak) in the topsoil of the basins of four major rivers in the Republic of Korea using spectroscopic technology and predicted the SOM content of each. The results of this study can be used to predict the organic and SOM components of topsoil using spectroscopic techniques as basic data for the construction of a soil database. A topsoil environment database could be utilized for mapping the distribution of topsoil organic components and could be filled by to remote technology that can measure topsoil components rapidly in the field.

## Figures and Tables

**Table 1:** Datasets used to develop and validate PLSR and SVR models for predicting SOM content.

	Total	GR ^(a)^			NR ^(b)^			YSR ^(c)^			HR ^(d)^		
		Total	WA ^(e)^	DWPZ ^(f)^	Total	WA	DWPZ	Total	WA	DWPZ	Total	WA	DWPZ
Calibration dataset	828	288	150	138	150	60	90	210	90	120	180	138	42
Prediction dataset	414	144	75	69	75	30	45	105	45	60	90	69	21

^(a)^ GR: Geum River, ^(b)^ NR: Nakdong River, ^(c)^ YSR: Yeongsan/Seomjin River, ^(d)^ HR: Han River, ^(e)^ WA: Waterfront Area, ^(f)^ DWPZ: Drinking Water Protection Zone.

**Table 2:** Soil organic matter (SOM) contents of topsoil samples from the basins of the four major rivers in the Republic of Korea.

Soil Sample		Count	SOM (g kg^−1^)			
			Min.	Max.	Ave.	Std.
Total		138	8.00	77.03	32.44	19.23
GR ^(a)^	Total	48	8.85	72.80	27.39	13.09
	WA ^(e)^	25	8.85	72.80	28.74	14.31
	DWPZ ^(f)^	23	10.40	55.08	25.91	11.12
NR ^(b)^	Total	25	11.14	75.46	52.61	20.70
	WA	10	11.14	73.91	38.82	18.77
	DWPZ	15	26.23	75.46	61.80	15.47
YSR ^(c)^	Total	35	10.82	77.03	37.83	17.93
	WA	15	10.82	77.03	39.06	19.56
	DWPZ	20	12.75	65.71	36.91	16.06
HR ^(d)^	Total	30	8.00	44.71	17.44	8.77
	WA	23	8.00	44.71	15.57	8.30
	DWPZ	7	13.71	33.76	23.58	6.58

^(a)^ GR: Geum River, ^(b)^ NR: Nakdong River, ^(c)^ YSR: Yeongsan and Seomjin River, ^(d)^ HR: Han River, ^(e)^ WA: Waterfront Area, ^(f)^ DWPZ: Drinking Water Protection Zone.

**Table 3:** The optimal SOM prediction model (PLSR or SVR) in the four major rivers.

Soil Sample		Model	Wavelength Range (nm)	Pre-Processing	Rc^2^	RMSEc (g kg^−1^)	Rv^2^	RMSEv (g kg^−1^)	Factor
Total		PLSR	400–1100	Normalization	0.657	11.23	0.630	11.66	8
		SVR	350–2500	1st derivative (20 nm)	0.716	10.55	0.678	11.12	-
GR	Total	PLSR	400–1100	Normalization	0.668	7.47	0.526	8.93	8
		SVR	350–2500	SNV	0.627	8.54	0.544	9.17	-
	WA	PLSR	350–2500	Normalization	0.680	8.10	0.556	9.60	5
		SVR	350–2500	1st derivative (20 nm)	0.649	9.56	0.531	10.38	-
	DWPZ	PLSR	350–2500	SNV	0.713	5.95	0.629	6.83	4
		SVR	350–2500	Normalization	0.794	5.33	0.735	5.97	-
NR	Total	PLSR	350–2500	-	0.766	9.81	0.659	11.96	7
		SVR	350–2500	1st derivative (20 nm)	0.735	10.54	0.662	11.84	-
	WA	PLSR	400–1100	1st derivative (20 nm)	0.912	5.57	0.746	9.55	9
		SVR	1100–2500	1st derivative (20 nm)	0.706	10.89	0.470	13.80	-
	DWPZ	PLSR	400–1100	-	0.806	8.28	0.703	10.29	7
		SVR	350–2500	2nd derivative (20 nm)	0.858	7.35	0.644	10.29	-
YSR	Total	PLSR	350–2500	MSC	0.829	7.31	0.678	10.21	8
		SVR	400–1100	1st derivative (15 nm)	0.708	9.96	0.675	10.42	-
	WA	PLSR	350–2500	SNV	0.912	5.79	0.826	8.17	7
		SVR	350–2500	1st derivative (15 nm)	0.838	9.03	0.682	11.76	-
	DWPZ	PLSR	350–2500	SNV	0.911	4.80	0.729	8.42	11
		SVR	350–2500	SNV	0.710	9.78	0.596	11.22	-
HR	Total	PLSR	350–2500	SNV	0.704	4.69	0.615	5.38	5
		SVR	350–2500	SNV	0.755	4.55	0.691	4.96	-
	WA	PLSR	350–2500	SNV	0.810	3.62	0.657	4.92	5
		SVR	350–2500	SNV	0.731	4.90	0.675	5.20	-
	DWPZ	PLSR	350–2500	SNV	0.877	2.31	0.694	3.72	6
		SVR	400–1100	SNV	0.689	3.82	0.559	5.76	-

## Data Availability

Not applicable.
